# The Miraprep: A Protocol that Uses a Miniprep Kit and Provides Maxiprep Yields

**DOI:** 10.1371/journal.pone.0160509

**Published:** 2016-08-03

**Authors:** Mira I. Pronobis, Natalie Deuitch, Mark Peifer

**Affiliations:** 1 Curriculum in Genetics and Molecular Biology, University of North Carolina at Chapel Hill, Chapel Hill, NC, United States of America; 2 Department of Biology, University of North Carolina at Chapel Hill, CB#3280, Chapel Hill, NC, United States of America; University of Manchester, UNITED KINGDOM

## Abstract

Plasmid purification is a basic tool of molecular biologists. Although the development of plasmid isolation kits utilizing silica spin columns reduced the time and labor spent on plasmid purification, achieving large plasmid DNA yields still requires significant time and effort. Here we introduce the Miraprep, a rapid protocol that allows isolation of plasmid DNA using commercial Miniprep kits, but with DNA yields comparable to commercial Maxiprep plasmid purifications. Combining ethanol precipitation with spin column purification, we created a DNA isolation protocol that yields highly concentrated plasmid DNA samples in less than 30 minutes. We show that Miraprep isolated plasmids are as stable as plasmids isolated by standard procedures, can be used for standard molecular biology procedures including DNA sequencing, and can be efficiently transfected into mammalian cells. This new plasmid DNA isolation protocol will significantly reduce time and labor without increasing costs.

## Introduction

Plasmid DNA isolation for cloning and protein expression has been in use for decades [1–3] and remains one of the most common methods used by molecular biologists. Plasmid purification involves two steps: bacterial lysis and DNA isolation. Bacterial lysis is often accomplished through alkaline lysis, a fast and effective way to separate protein and genomic DNA from small plasmids [3]. For plasmid DNA isolation, however, several methods are available.

Commercial plasmid preparation kits decrease time and labor. Plasmid DNA isolation via silica spin columns is currently the standard method [4, 5]. These are thought to depend on the ability of “chaotropic salts” to dehydrate DNA, allowing the DNA to bind reversibly to the silica via its phosphate groups [6, 7]. However, high-yield kits require a large time investment when compared to lower-yield kits (Table 1, Maxiprep vs. Miniprep). Additionally, DNA yield is restricted by column size, since silica columns bind limiting amounts of plasmid DNA [4]. Thus, manufacturers traditionally advertise their plasmid isolation kit by the maximal amount of DNA that can be bound by a column (Table 1). Howeverowever it is important to note that these estimates do not take into account plasmid size, type or copy number, or growth conditions of the bacterial strain, or the bacterial strain itself. All these factors are known to influence DNA yield.

**Table 1:** Comparison of commercial plasmid preparation methods from three manufacturer’s (GeneJet, Qiagen, and GenElute) with the Miracle-prep.

QIAGEN*	Bacteria culture (ml)	Max. column capacity (μg)	**Average yield in n = exp.(μg)**	Elution volume (μl)	Time (min)	Costs per μg DNA
Spin Miniprep	1–5	20–25	23±5 (n = 3)	50–60	< 30	6.2 cents
Midiprep	25–100	~ 100	N.D.	variable	150	9.8 cents
Maxiprep	100–500	~ 500	237±226 (n = 6)	variable	160	4.4 cents
GeneJET*	Bacteria culture (ml)	Max. column capacity (μg)	**Average yield in n = exp.(μg)**	Elution volume (μl)	Time (min)	Costs per μg DNA
Miniprep	1–5	20–30	36±10 (n = 3)	35–50	< 14	3.6 cents
Midiprep	50	200–280	N.D.	250–350	60	2.3 cents
Maxiprep	250	770–1100	592±243 (n = 4)	700–1000	60	1.4 cents
						
GenElute*	Bacteria culture (ml)	Max. column capacity (μg)	**Average yield in n = exp.(μg)**	Elution volume (μl)	Time (min)	Costs per μg DNA
Miniprep	1–5	20	**15**±9.7 (n = 4)	50–100	< 30	7.5 cents
**Miracle-prep**	Bacteria culture (ml)	Max. column capacity (μg)	**Average DNA yield in n = 25 exp. (μg)** **	Elution volume (μl)	Time (min)	Costs per μg DNA**
5x	50	N.A.	392±200	150–175	< 30	Average = 1.4 cents

* Based on manufacturer’s recommendations.

**GeneJET Miniprep Kit was used.

N.D. Not done. N.A. Not applicable as column capacity was no longer a limiting factor.

Plasmid DNA precipitation via ethanol or isopropanol, which disrupts charge screening by water and allows positive ions in the solution to interact with DNA phosphate groups, was commonly used prior to development of silica columns. Although low in cost, the precipitation, centrifugation, and pellet washing steps require significant time even for small-scale preparations, and high yield kits require larger scale bacterial cultures and even more time. Intriguingly, the binding and washing buffers of some commercial silica spin columns also include alcohols (e.g. ethanol in the QIAprep Miniprep PE wash buffer [8]), which like chaotropic salts can increase DNA interaction with the silica matrix.

Here we report a modified protocol to isolate high plasmid DNA yields at significantly reduced time and labor, which we call the Miraprep.

## Material and Methods

### Miraprep protocol

A 50 ml bacterial culture was inoculated in appropriate selective media and incubated on a shaker at 250 rpm at 37°C overnight. On the next day the bacterial culture was transferred into a 50 ml tube and spun at 4000xg at 4°C for 10 minutes. The supernatant was discarded and the pellet was resuspended in 2 ml resuspension buffer with 50 μg/ml RNase (ThermoFisher #EN053) freshly added. 2 ml of lysis buffer was added to the bacterial suspension and the tube was inverted 3–4 times. It was then incubated for 3 minutes at room temperature. 2 ml of neutralization buffer was added and the tube was inverted 3–4 times. The bacterial lysate was quickly distributed into 1.5 ml centrifuge tubes (~4 tubes) by pouring, not pipetting, and spun at 13,200xg at room temperature for 10 minutes. Next, the supernatants were collected in a 15 ml tube and the pellets discarded. 1x volume of 96% ethanol (~5 ml) was added to the supernatant and mixed thoroughly for 5 seconds. The sample-ethanol mix was loaded onto 5 spin-columns in three sequential ~700μl aliquots, and the column were spun for 30 seconds at 13,200xg after the addition of each aliquot. After each spin the flow-through was discarded. These steps were repeated until the entire sample was passed through the spin-columns. The columns were washed two times with 500 μl wash buffer, spun after each wash at 13,200xg at room temperature for 30 seconds, and the flow-through was discarded. The empty columns were then spun one last time at 13,200xg at room temperature for 1.5 minutes to remove any residual wash buffer. After this, the old collection tube was discarded and each column was put onto a new tube. 30–35 μl of distilled water or elution buffer was added to the column, which was incubated for 2 minutes at room temperature, and spun at 13,200xg for 2 minutes to elute the DNA from columns. The eluted DNA from all 5 columns was combined in one tube (~175 μl). After measuring the DNA concentration the samples were stored at -20°C.

### Plasmids

The 3 kb plasmid was pmaxGFP (Lonza, Basel, Switzerland), which encodes standard GFP (GenBank L29345.1). The 8 kb and 14 kb plasmids have the same backbone, pEGFP-N1 (Clontech, Mountain View, CA, USA), and encode fly Adenomatous polyposis coli2 (APC2; GenBank NM_058082.3) or human APC1 (GenBank NM_001127511.2), respectively [9].

### Plasmid isolation kits

Mirapreps and Minipreps were conducted using either GeneJet from Thermo Fisher (Waltham, MA, USA), QIAGEN (Hilden, Germany), or GenElute from Sigma (St. Louis, MO, USA). The Endofree Maxiprep kit from QIAGEN or the GeneJet Maxiprep kit from Thermo Fisher were used for Maxi preparations. The centrifugal filter ‘Mini Filter Spin Column’ from Norgen Biotek (ON, Canada) was used to test whether a filter would retain plasmid DNA by filtration. These filter columns contain a 0.22 μm nylon membrane filter with a 2 ml capped collection tube. We proceeded through the Miraprep protocol up to ethanol addition using the buffers from the GenElute Miniprep kit (Sigma). After ethanol addition, the sample was added to the top of the filter and spun 30 sec at 13,200xg, washed as described in the Miraprep protocol above, and DNA was recovered from the top of the filter in 50 μl of ddH2O.

### Antibodies, immunofluorescence and immunoblotting

For Immunofluorescence staining the primary antibody was anti-β-catenin from BD Transduction Laboratories (San Jose, CA, USA, used at 1:1000), and the secondary antibody was the Alexa 647-labeled anti-mouse antibody from Invitrogen (Carlsbad, CA, USA, used at 1:1000). For immunoblotting SW480 cells were lysed directly in SDS loading buffer, samples electrophoresed on 8% SDS gels and blotted to nitrocellulose. The primary antibody was anti-GFP from Abcam (Cambridge, MA, USA, used at 1:10,000), and the secondary antibody was HRP-labeled anti-rabbit (Pierce, Rockford, IL, USA, used at 1:50,000).

### Cell culture

The SW480 cell line was obtained from the ATCC (CCL 228) via the UNC-Chapel Hill Lineberger Comprehensive Cancer Center Tissue Culture Facility. SW480 cell culture conditions and the transfection protocol were as described in [9]. Lipofectamine 2000 (Invitrogen, Grand Island, NY, USA) and 2 ug DNA /well (in a six well plate) were used for transfections.

### Plasmid DNA comparison to DNA ladder

The gel band analyzer tool from ImageJ software was used to measure the intensity of bands of plasmid DNA and the DNA ladder.

## Results

### Adding ethanol after the neutralization step increases yields from commercial silica spin column-based DNA Miniprep kits

**Fig 1:**
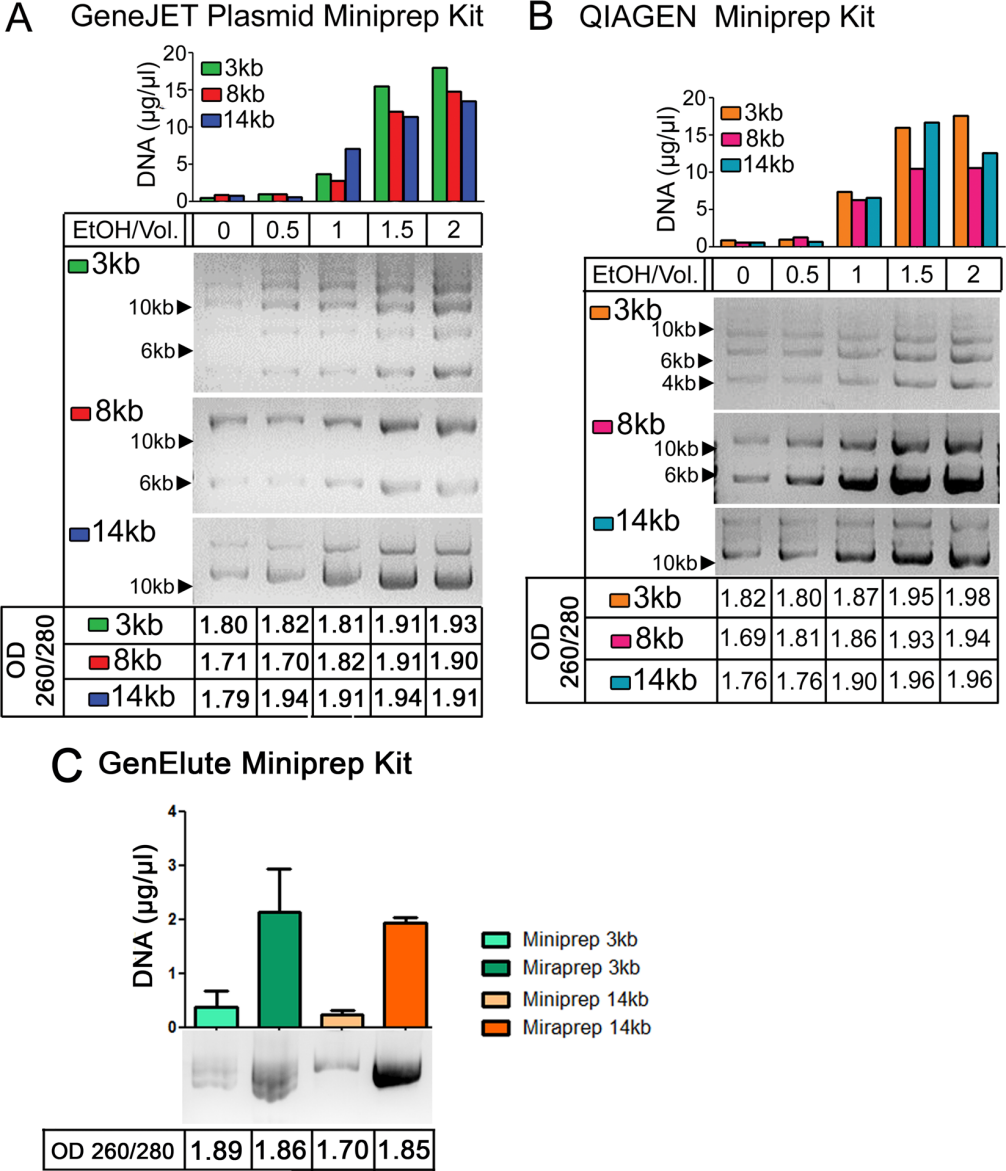
Addition of Ethanol leads to increased plasmid DNA yield. (A) DNA plasmid preps of the indicated plasmids with different concentrations of ethanol. Top: DNA concentration as assessed by OD260, middle: 2 μl of each sample was electrophoresed on an agarose gel and visualized by ethidium bromide staining, bottom: OD260/280 ratio. The GeneJET Plasmid Miniprep kit was used. (B) As in (A) but the Qiagen Miniprep kit was used. (C) Plasmid preparations with the GenElute kit, comparing either the standard Miniprep procedure or the Miraprep (using 1x volume of ethanol). The Mirapreps in (C) included fresh addition of RNase (50 μg/ml) as in the final Miraprep protocol, and values are the average of two experiments, showing mean and standard deviation.

Commercial Maxiprep kits require a large time investment. This led us to explore alternate methodologies to reduce the preparation time, but still maintain high yields of DNA. Ethanol can act to dehydrate DNA, which is predicted to increase interaction with silica, and for this reason a number of commercial kits include alcohols in the binding or wash buffers. We thus explored whether adding ethanol after the alkaline lysis and neutralization steps and before loading on the column might increase yields of commercial Miniprep kits. We first explored how varying the ethanol concentration affected both yield and DNA purity. We examined three different size high copy plasmids: a 3 kb plasmid, pmaxGFP, which encodes standard GFP, and 8 kb and 14 kb plasmids sharing the same backbone, pEGFP-N1, and encoding *Drosophila* Adenomatous polyposis coli2 (APC2) or human APC, respectively [9] (the plasmid suppliers estimate backbone copy number of 500/cell). We initially tried two different commercial Miniprep kits: GeneJet and QIAGEN. DNA yield of all three size plasmids increased significantly when 1x volume of ethanol was used, while 1.5x or 2x volumes of ethanol provided the highest apparent DNA yield, as assessed by the OD260 (Fig 1A and 1B, top). We next tested DNA purity by calculating the OD260/280 ratio (Fig 1A and 1B, bottom). Pure DNA has a ratio of 1.8, and thus a ratio of 1.75–1.95 is generally considered to be a good DNA preparation [10]. A ratio >1.95 indicates RNA contamination while a ratio <1.7 indicates protein contamination. The 1x volume Miraprep samples had OD260/OD280 ratios of 1.81–1.91, while samples with 1.5x volumes of ethanol were >1.91, suggesting RNA or other types of contamination in the latter. When DNA yields were assessed by gel electrophoresis and comparison of the plasmid bands to known DNA standards, 1x volume Miraprep samples had DNA concentrations consistent with their OD260, similarly to what we saw with Miniprep or Maxiprep samples (see below), while 1.5x volume Miraprep samples were inconsistent relative to their DNA amount determined by gel electrophoresis (data not shown). Thus a 1x volume of ethanol for DNA precipitation was selected. We further verified that this protocol using 1x volume of ethanol increased DNA yields when using a third commercial spin column Miniprep kit, Sigma GenElute (Fig 1C).

**Fig 2:**
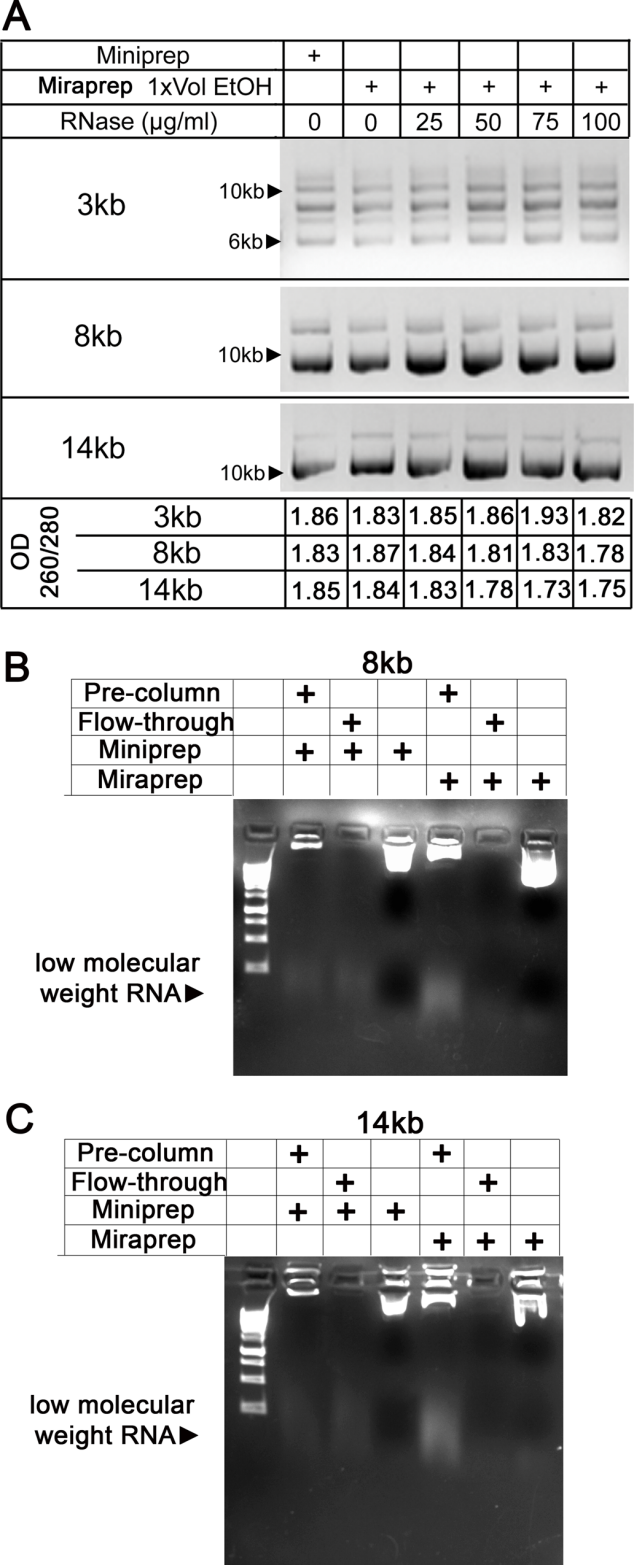
Different RNase concentrations do not reduce DNA yield in Miraprepped samples and Miraprep is not significantly contaminated by low molecular weight RNA. (A) Standard Miniprep, or Miraprepped plasmids prepared using 1x volume of ethanol, were treated with indicated RNase concentration, added freshly into the resuspension buffer before beginning the procedure. Top: 0.4 μg was electrophoresed on an agarose gel. DNA concentration only varied slightly when RNase was freshly added. Bottom: OD260/280 ratio. (B,C) Testing for low molecular weight RNA in Miniprep and Miraprep samples, respectively. (B) Miraprep and Miniprep samples of the 8 kb plasmid contain little or no small molecular weight RNA. Pre-column = after alkaline lysis, Flow-through = flow-through of spin column, Final lane in each set is eluted plasmid. 10 μl of pre-column and flow-through samples were loaded, while 2 μl were loaded of Miniprep or Miraprep samples. (C) Miniprep and Miraprep samples of the 14 kb plasmid have little to no low molecular RNA present. Loading same as described in (B).

The differences in OD260/OD280 ratios seen with different amounts of ethanol addition suggested the possibility of RNA contamination. Consistent with this, when we compared older Miniprep kits, in which RNase was added to the resuspension buffer several weeks earlier, with new Miniprep kits, where the manufacturer’s RNase was freshly added, DNA yields as assessed by OD260 sometimes were higher than those determined by comparison to DNA markers of known concentrations analyzed via agarose gel electrophoresis (data not shown). This suggested that the RNase activity in the resuspension buffer might become depleted over time and become insufficient to decrease RNA levels efficiently. We thus explored whether fresh RNase addition might alleviate this issue. Fresh RNase addition into the Miraprep resuspension buffer at 0–100 μg/ml did not reduce DNA yields, as assessed by gel electrophoresis (Fig 2). Thus to achieve consistent and effective RNA depletion in Miraprep samples the final protocol includes adding fresh 50 μg/ml RNase to the resuspension buffer before each preparation. We verified that our modified Miraprep eliminated the low-molecular weight RNAs present in the initial preparation as well as they were removed by a standard Miniprep. To do so, we examined by gel electrophoresis the initial cell lysate after alkaline lysis and the spin to remove cellular debris and high molecular weight DNA (Fig 2B and 2C Pre-column), the initial flow-through (Fig 2B and 2C Flow-through), and the final eluted plasmid DNA (Miniprep or Miraprep). While low-molecular weight RNAs were clearly present in the initial lysate, they were at very low to undetectable levels in the eluted plasmid DNA, suggesting that our estimated Miraprep plasmid yields are not significantly altered by contaminating small molecular weight RNAs (this is also consistent with the efforts below to estimate plasmid yield directly by gel electrophoresis and comparison to known DNA standards).

**Fig 3:**
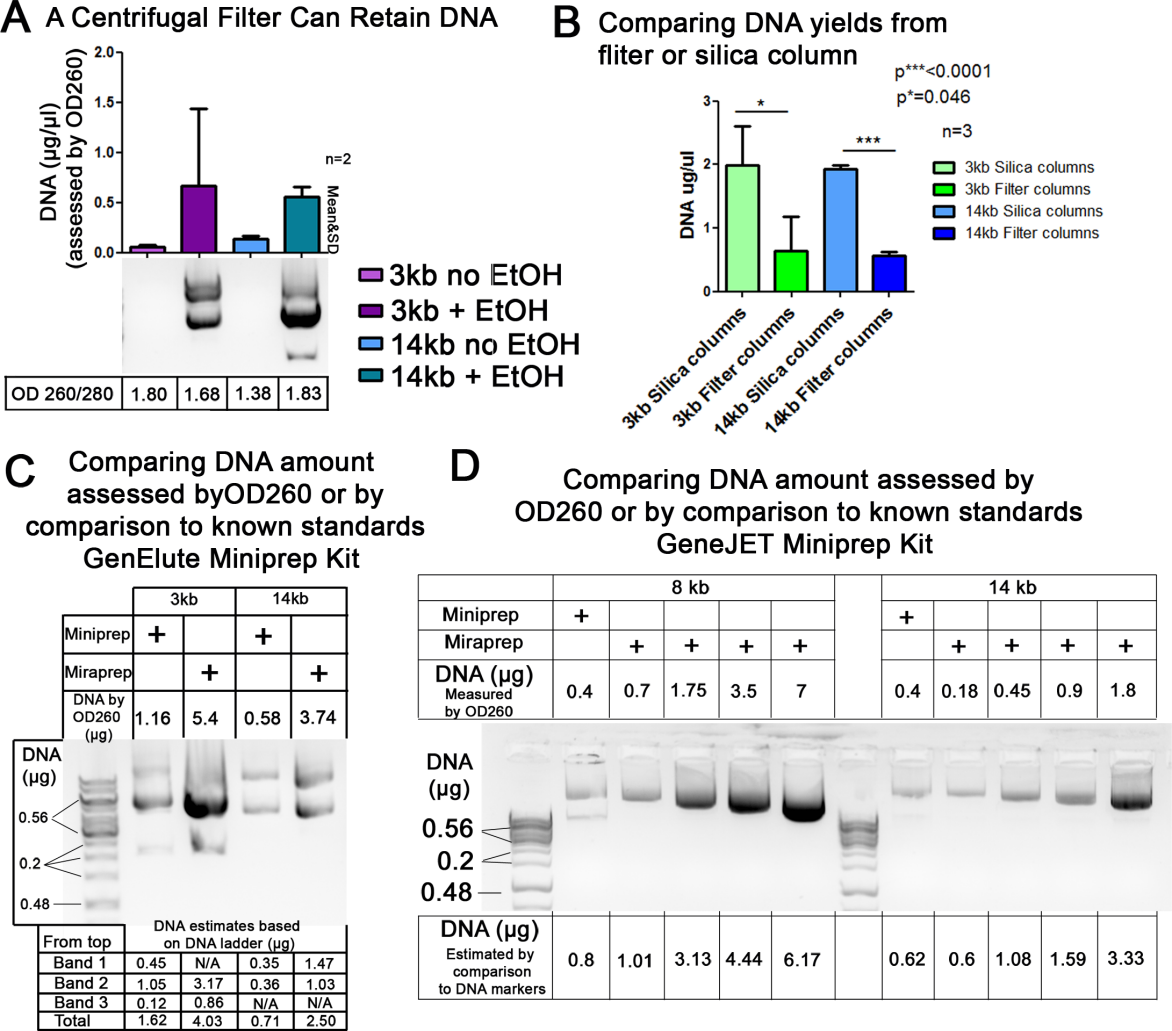
Testing whether the columns might act as a filter and verifying DNA yields using comparison to known DNA standards. (A) To determine whether silica spin columns might capture DNA by acting as filters, the Miraprep procedure was followed through the neutralization step with 1x volume of ethanol added or no ethanol added as a control, and then the sample was passed over a simple centrifugal filter (pore size 0.22 μm), the filter was washed following the Miraprep protocol, DNA was eluted from the top surface of the filter, and electrophoresed on an agarose gel. DNA was only recovered after ethanol addition. (B) Silica columns are more efficient than centrifugal filters in capturing plasmid DNA. Comparison of DNA yields using silica columns or centrifugal filter columns; from three independently conducted Mirapreps. (C,D) Standard Miniprep plasmids, or Miraprep plasmids prepared using our final protocol using 1x volume of ethanol, were electrophoresed on an agarose gel and amounts compared to known DNA standards (Thermo Scientific GeneRuler 1 kb DNA Ladder #SM0312 (0.5 μg/μl)). 2 μl DNA plus 5 μl loading buffer were loaded in each lane. (C) GenElute kit. (D) GeneJET kit. Above each gel is the DNA amount calculated from OD260 and below the gel estimates from comparison to DNA markers of known amounts. Image J was used to quantify DNA band intensities in (C,D). DNA amounts calculated by both methods were comparable.

One possible mechanism by which the Miraprep procedure could increase DNA yields was that the ethanol addition led to DNA precipitation and that the silica column acted, at least in part, as a filter to capture the precipitated DNA. To determine whether this was plausible, we went through our procedure to the neutralization step, added 1x volume of ethanol or no ethanol as a control, and then ran the sample over a simple centrifugal filter (pore size 0.22 μm). We then eluted DNA from the top surface of the filter. Consistent with the idea that the column acts as a filter, we were able to effectively capture DNA on the centrifugal filter only from samples where ethanol was added (Fig 3A). However, DNA recovery from the centrifugal filter was not as effective as that from the silica columns (Fig 3B), suggesting that the columns may act as more than just a filter.

### Comparison of the final Miraprep protocol to commercial silica spin column DNA preparations

The final protocol for large scale Mirapreps was tested with GeneJet, QIAGEN, and Sigma GenElute spin column Miniprep kits, using the resuspension, lysis, neutralization and wash buffers provided (a step-by step protocol is provided in S1 File). The final protocol allows DNA to be efficiently isolated from relatively small bacterial cultures (50 ml) in less than 30 minutes (Table 1). Increased yields were noted with the kits from each of the manufacturers. We further verified that in this final protocol, DNA yields as assessed by OD260 were consistent with those determined by comparison to known DNA standards after agarose gel electrophoresis (Fig 3C and 3D). In 25 Mira-preparations we recorded an average DNA yield of 392±200 μg. Our Minipreps produced yields similar to the maximum column capacity indicated by the manufacturers. Thus the Miraprep had a much higher yield on average than the maximum column capacities of Mini-preps (Table 1). We also compared the yields from Mirapreps with those from two commercial Maxiprep kits, using both the manufacturer’s maximum column capacities and our own lab’s typical yields using these kits as benchmarks. We found that the Miraprep produced yields in range of the commercial available Maxiprep kits (Table 1), but with significant time savings.

### DNA prepared using the Miraprep protocol is stable and of sufficient purity for DNA sequencing and mammalian cell transfection

**Fig 4:**
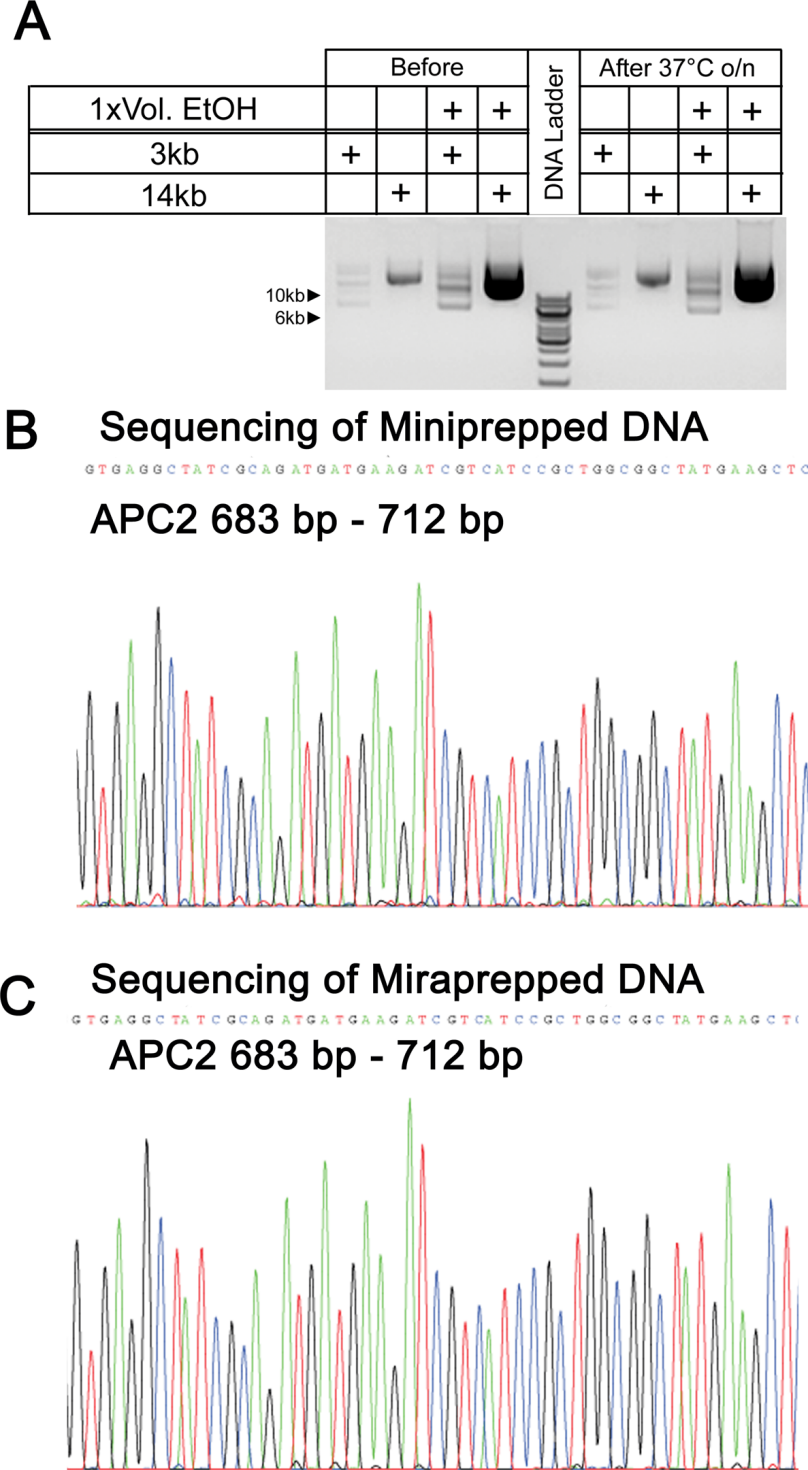
Miraprepped samples are stable and can be used for sequencing. (A) Plasmids prepared using the Miraprep protocol with 1x volume of ethanol+50 μg/ml RNase are as stable as commercial Miniprepped plasmids after incubation overnight at 37°C. 2 μl of Mira- or Miniprepped samples were loaded. (B) Sequencing reaction of a Miniprepped APC2 (8 kb) plasmid—the sequence from 683 base pairs (bp)—712 bp is shown. 0.7 μg of DNA was used for the sequencing reaction (C) Sequencing of Miraprepped APC2 plasmid. 0.7 μg of DNA was used, and the same region as in (B) is shown.

We next verified that the DNA produced by the Miraprep protocol is of sufficient purity to be used in standard molecular and cell biology procedures. We first examined plasmid DNA stability. Similar ratios of circular/supercoiled plasmids were detected in both Miniprepped and Miraprepped samples (Figs 2, 3C and 3D), suggesting the Miraprep protocol does not lead to DNA nicking. Mini- and Miraprepped samples also did not show any DNA degradation after incubation overnight at 37°C (Fig 4A). Thus plasmids prepared by the Miraprep protocol are as stable as samples prepared using the regular Miniprep isolation protocol. We also verified that Miraprep plasmids can be used for DNA sequencing. Sequencing quality from Miraprepped plasmids was similar to that from commercial Miniprepped plasmids (Fig 4B and 4C).

**Fig 5:**
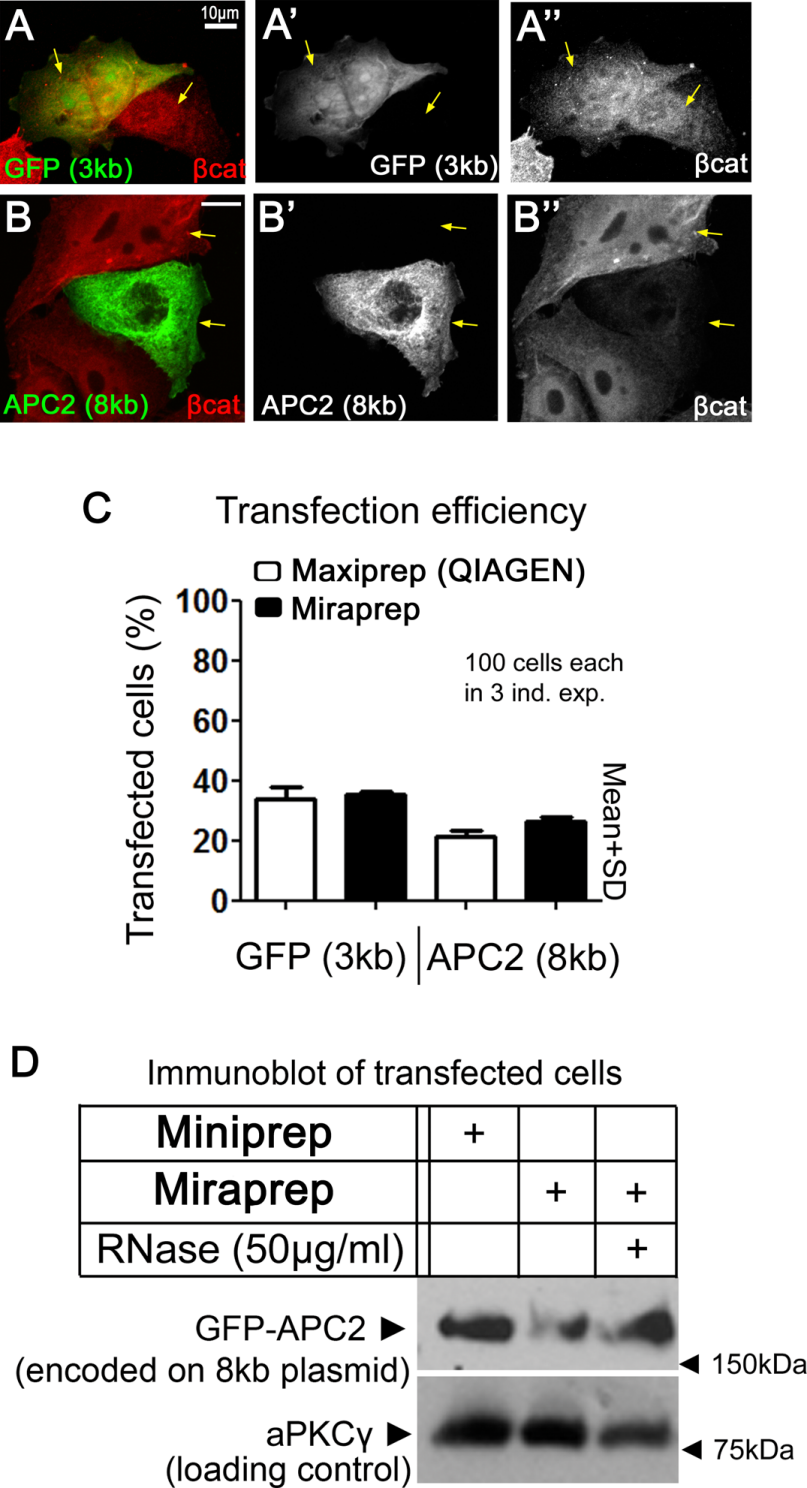
Miraprepped plasmids can be effectively used to transfect human tissue culture cells. (A) Immunofluorescence of human SW480 cells transfected with a plasmid encoding GFP (3 kb; Miraprepped using 1x volume of ethanol+50 μg/ml RNase) and stained for β-catenin via antibody. SW480 cells have high levels of the Wnt transcriptional co-activator β-catenin due to a mutation in one of its key negative regulators, APC. (A’) GFP is uniformly distributed throughout transfected cells. (A”) Expression of GFP does not alter β-catenin levels—arrows compare a transfected and an untransfected cell. (B) Immunofluorescence of SW480 cells transfected with a plasmid encoding GFP-tagged *Drosophila* APC2 (8 kb; Miraprep using 1x volume of ethanol+50 μg/ml RNase). (B’) APC2 is uniformly distributed in the cytoplasm. (B”) Fly APC2 is able to reduce β-catenin levels, thus compensating for the mutation of the endogenous human APC in the SW480 cells. (C) Transfection efficiency into SW480 cells is similar for Miraprepped samples (using 1x volume of ethanol+50 μg/ml RNase) and those transfected with DNA prepared via the standard Qiagen Maxiprep procedure. 2 μg of plasmids encoding GFP (3 kb) or GFP-tagged Drosophila APC2 (8 kb) were transfected using Lipofectamine 2000. 100 cells were counted in each of three independent experiments. (D) Immunoblot analysis of transfection efficiency. 3 conditions were tested: DNAs prepared by Miniprep (GeneJET), Miraprep (using 1x volume ethanol), and Miraprep (using 1x vol+50 μg/ml RNase). All led to roughly equal levels of protein expression. Lipofectamine 2000 and 2 μg of plasmid DNA were used. Cells were directly lysed in SDS-loading buffer. aPKCγ was used as the loading control.

Maxiprep plasmids are commonly used for mammalian cell transfections, which require high purity DNA. To test whether Miraprep purified plasmids were suitable for tissue culture transfections we used human SW480 colon cancer cells. These cells have constitutively active Wnt signaling due to a mutation of endogenous APC [11]. The tumor suppressor APC is a key negative regulator of Wnt signaling and down regulates the protein levels of β-catenin, the transcriptional co-activator of Wnt target genes. With the loss of APC, β-catenin is no longer targeted for degradation and accumulates in the cytoplasm and nucleus. Thus SW480 cells have high levels of β-catenin. To test transfection efficiency of Miraprep samples, we transfected two different sized plasmids, a 3 kb plasmid encoding GFP, which does not alter β-catenin levels, and an 8 kb plasmid encoding *Drosophila* GFP-APC2, which can downregulate β-catenin levels in SW480 cells [9, 12]. GFP and GFP-APC2 were each detected in SW480 cells (Fig 5A, 5A’, 5B and 5B’) and localized as expected [12]. While β-catenin levels in GFP-transfected cells were similar to untransfected cells (Fig 5A and 5A”, compare cells indicated by arrows), APC2-transfected cells had decreased β-catenin (Fig 5B and 5B”, compare cells indicated by arrows). To compare transfection efficiency, we counted 100 cells each in 3 independent experiments and compared the percentage of transfected cells as assessed by GFP fluorescence (Fig 5C). We found no statistically significant difference between Maxi- and Miraprepped samples. We also determined transfection efficiency by examining expression of the encoded proteins. Immunoblotting revealed no significant difference in APC2 protein expression in SW480 cells transfected with either Mini- or Miraprepped (+RNase) DNA (Fig 5D). Thus Miraprep plasmids can be efficiently transfected into mammalian cells, and plasmid-encoded genes are transcribed and translated into functional protein.

## Discussion

Plasmid purification is a basic tool of molecular biologists. Although the development of plasmid isolation kits with silica spin columns reduced time and effort spent on plasmid purifications, high yields of plasmid DNA still require significant time and labor (Table 1). Our modified rapid plasmid DNA isolation protocol, the Miraprep, provides DNA yields comparable to commercial Maxiprep isolation kits, but at a significantly reduced time investment (less than 30 minutes) and without increasing costs (Table 1).

The Miraprep involves addition of ethanol to the DNA early in the protocol, after the neutralization step and before adding to the spin column. Our data are consistent with the idea that this protocol combines advantages of both silica based spin columns and ethanol precipitation: (1) Upon addition of ethanol the entire plasmid DNA pool in the sample appears to become insoluble and precipitates out, and (2) DNA precipitates are then captured by the silica column. Under these circumstances, DNA yield is not limited by the column’s maximum DNA binding capacity (a Miniprep column holds only 20 μg). We suspect that the silica gel may be acting in part as a filter, a hypothesis supported by our ability to retain and recover DNA from a simple centrifugal filter (Fig 3A).

Our protocol yields highly concentrated plasmid DNA samples in less than 30 minutes (Table 1). We assessed yield in 25 different Mirapreps of multiple different plasmids, obtaining yields of 392±200 μg plasmid DNA. This is very similar to Maxiprep DNA yields we routinely obtain using Maxiprep kits of both manufacturers (Table 1), and our maximum yield (770 μg among the 25 experiments) met or exceeded the theoretical maximum yields of the Maxiprep kits. Our protocol also reduced the time involved significantly. While Maxipreps take between 60–160 min, the Miraprep protocol can be conducted in less than 30 minutes (Table 1). Finally, plasmid DNAs isolated by our Miraprep protocol are similar in DNA quality (evaluated by OD260/OD280 ratio and gel electrophoresis), stability, transfection efficiency, and protein expression levels as plasmids isolated by standard Mini- or Maxi-preparations.

## Supporting Information

S1 File Step-by-step Miraprep protocol. (DOCX)
