# Supplementary material for: The Miraprep: A Protocol that Uses a Miniprep Kit and Provides Maxiprep Yields
Source: PLoS One. 2016 Aug 3;11(8):e0160509. doi: 10.1371/journal.pone.0160509 (PMC4972323; doi:10.1371/journal.pone.0160509)
Supplement: S1 File — (DOCX) [file pone.0160509.s001.docx]

**S1 File. Step-by-step Miraprep protocol.**

**Protocol Title: Miracle-prep (Miraprep)**

**PROTOCOL FOR: Plasmid DNA isolation**

**Manuscript Title: The Miraprep: A protocol that uses a Miniprep kit and provides Maxiprep yields**

**Authors Mira I. Pronobis^1^, Natalie Deuitch^2^ and Mark Peifer^1,2^***

**REAGENTS**

GeneJet Plasmid Miniprep Kit (Thermo Scientific), or

QIAprep Spin Miniprep Kit (Qiagen)

**PROCEDURE**

1. Set up a 50 ml bacterial culture in appropriate selective media and incubate on a shaker (250 rpm) at 37°C overnight.

2. Transfer bacteria to a 50 ml tube and spin at 4000xg at 4°C for 10 min.

3. Discard supernatant and resuspend pellet in 2 ml resuspension buffer with 50 ug/ml RNase freshly added.

4. Add 2 ml of lysis buffer, invert 3-4 times and incubate for 3 min at RT.

5. Add 2 ml of neutralization buffer and invert 3-4 times.

6. Distribute lysate into 1.5 ml Eppendorf tubes (~4 tubes) by pouring, not pipetting.

7. Spin at 13,200xg at RT for 10 min.

8. Collect supernatants in a 15 ml tube and discard pellets.

9. Add 1x volume of 96% ethanol (~5 ml).

10. Mix thoroughly for 5 sec.

11. Load the sample-ethanol mix onto 5 spin-columns in three sequential ~700 µl aliquots--after the addition of each aliquot, spin the column 30 sec at 13,200xg.

12. Discard flow-through.

13. Repeat step 11 until the entire sample is run through the spin-columns.

14. Wash with 500μµl washing buffer and spin at 13,200xg at RT for 30 sec.

15. Discard flow-through.

16. Repeat step 14-15.

17. Do a final spin at 13,200xg at RT for 1.5 min.

18. Discard the old tube and put the column onto a new tube.

19. Add 30-35μµl of ddH2O and incubate for 2 min at RT.

20. Spin at 13,200xg for 2 min to elute the DNA from column.

21. Combine the eluted DNA from all 5 columns in one tube (~175μµl).

22. Measure DNA concentration.

23. Store samples at -20°C.
